# Implantation of a Canine Total Ankle Replacement Prosthesis Using a Lateral Surgical Approach is Accurate and Leads to a Stable Joint

**DOI:** 10.1055/a-2654-8080

**Published:** 2025-07-25

**Authors:** Michelle M. Zingel, Laurent P. Guiot, Denis J. Marcellin-Little, Tanya C. Garcia, Jennifer L. Hubbard

**Affiliations:** 1 Surgery Department, Veterinary Specialty Center of Seattle, Lynnwood, Washington, United States; 2 Bone and Joint Center, ACCESS Specialty Animal Hospital, Culver City, California, United States; 3 Department of Surgical and Radiological Sciences, School of Veterinary Medicine, University of California, Davis, Davis, California, United States; 4 JD Wheat Veterinary Orthopedic Research Laboratory, School of Veterinary Medicine, University of California, Davis, Davis, California, United States; 5 Surgery Department, Veterinary Centers of America (VCA) Animal Specialty and Emergency Center, Los Angeles, California, United States

**Keywords:** total ankle replacement, surgical approach, dog

## Abstract

**Objective:**

This study aimed to determine if canine total ankle replacement (cTAR) can be performed using a lateral surgical approach by comparing implant orientation, limb orientation and tarsocrural stability after implantation using a lateral or medial approach.

**Study Design:**

Ten cadaveric limbs from five large-breed dogs were implanted with a cTAR prosthesis using a medial or a lateral approach. Caudocranial and mediolateral radiographs were obtained. Joint orientation, limb orientation, angular tarsocrural stability (varus and valgus laxity) and rotational tarsocrural stability (internal and external rotational laxity) were measured before and after implantation and compared. Polar gaps around cTAR components were measured.

**Results:**

Before implantation, mean valgus laxity was 1.8 degrees larger in limbs which were implanted with a cTAR prosthesis using a lateral approach than in limbs implanted using a medial approach. After a lateral approach, mean valgus laxity was 4.4 degrees larger (7.2 degrees) than before (2.8 degrees), and mean external rotational laxity was 5.4 degrees larger (10.7 degrees) than before (5.3 degrees). After a medial approach, mean external rotational laxity was 6.7 degrees larger (11.6 degrees) than before (4.9 degrees). The mean angular laxity was 6.0 degrees larger after a lateral approach (15.5 degrees) than a medial approach (9.5 degrees). Significant differences among other measurements collected after a lateral or medial approach were not identified.

**Conclusion:**

A cTAR prosthesis can be implanted using a lateral approach and result in a properly oriented tarsocrural joint that is rotationally stable and has slight angular laxity.

## Introduction

In dogs, tarsocrural joint osteoarthritis can result from conditions such as trauma, osteochondritis dissecans and immune-mediated disease. 1 2 Tarsocrural osteoarthritis often causes pain, lameness and a loss of function. 3 Traditional management options for tarsocrural osteoarthritis include the administration of pain medications and pantarsal arthrodesis. 4 5 A canine total ankle replacement (cTAR) prosthesis is undergoing a limited clinical evaluation. 6 In humans, total ankle replacement is an established therapeutic option for end-stage tarsocrural osteoarthritis performed in approximately 10,000 patients per year. 7 8 Although the anterior (cranial) midline approach is widely used to implant prostheses in humans, a lateral transfibular surgical approach to total ankle replacement has been used to reduce iatrogenic neurovascular damage and improve patient outcomes. 9 The current guidelines for cTAR implantation are to use a medial surgical approach with osteotomy of the medial malleolus. 6 However, a lateral approach using a fibular osteotomy and retraction could potentially offer advantages, similar to the lateral approach used for human total ankle replacement and for the implantation of canine total elbow replacement prostheses. 10 It could facilitate exposure, help preserve neurovascular structures on the medial aspect of the joint and avoid dissection of the medial aspect of the joint in patients with fibrosis or orthopaedic implants.

The purpose of this pilot study was to evaluate joint alignment, limb alignment and joint stability in paired cadaver limbs when cTAR implantation was performed using a lateral approach compared with a medial approach. We hypothesized that using a lateral approach for cTAR would result in implantation accuracy and joint stability similar to those obtained with cTAR implanted using a medial approach. Angular stability (frontal plane laxity) was evaluated by measuring varus and valgus with the tarsocrural joint in extension. Rotational stability (transverse plane laxity) was evaluated by measuring internal and external rotation with the tarsocrural and stifle joints at 90 degrees.

## Methods

### Sample

Cadavers were ethically sourced (Skulls Unlimited, Oklahoma City, OK). Cadavers were thawed to room temperature (∼17 °C) for 24 hours. A cTAR was implanted in the right pelvic limb using a randomly selected medial approach or lateral approach (Excel version 16.94, Microsoft, Redmond, WA). The left paired limb received a cTAR prosthesis implanted using the alternative approach. Limbs with evidence of an orthopaedic problem and limbs with bones deemed too small or large to undergo total ankle replacement were excluded from the study.

### Radiographic Assessment

A 1.6-mm-diameter Kirschner wire was inserted into the tibia adjacent to and distal to the tibial tuberosity in a craniocaudal orientation. The wire was trimmed, leaving 3 cm protruding from the bone surface. The wire served as a radiographic rotational alignment marker. The operated limbs were radiographed using digital radiography before and after total ankle replacement implantation. The eight radiographic views included three caudocranial (CC) views, two mediolateral (ML) views and three proximodistal horizontal beam (PDHB, skyline) views of the tibia. All views included the stifle joint, tibia, tarsus, pes and a 100-mm-long calibration marker. The marker was placed along the distal portion of the tibia and was parallel to the flat panel detector. The three CC views included a view without stress, a view with varus stress and a view with valgus stress. Dogs were positioned in sternal recumbency and secured using sandbags, foam blocks and tape. The X-ray beam was centred over the tibial diaphysis. The medial aspect of the calcaneus bisected the intermediate ridge of the tibial cochlea. 11 For the varus stress CC view, the lateral aspect of the pes was stressed in the frontal plane using a nylon rope, secured at the distal aspect of the metatarsal bones and tensioned with a 4.5-kg weight using a vertical pulley. A traction of 4.5 kg was selected to remove the slack from the joint without damaging the collateral ligaments. 12 Counterpressure was applied to the medial aspect of the distal portion of the femur, the stifle and the proximal portion of the tibia. For the valgus stress CC view, the medial aspect of the pes was similarly stressed while counterpressure was applied to the lateral aspect of the distal portion of the femur, the stifle and the proximal portion of the tibia. For the two ML views, dogs were positioned in lateral recumbency with the limb being radiographed lowest and the tarsus parallel to the table. The X-ray beam was centred on the talus. The medial and lateral talar ridges were superimposed. One ML view was acquired with the stifle and tarsocrural joints held at approximately 90 degrees, and a second ML view was acquired with the stifle and tarsocrural joints in maximal flexion. The three PDHB views were made with the dogs positioned in dorsal recumbency in a V-trough. The stifle and tarsus were placed at approximately 90 degrees. The tibia was stabilized using a toothed reduction handle with threaded pin (DePuy Synthes, Paoli, PA) secured to the medial aspect of the proximal metaphysis, in the location where a jig pin is placed during tibial plateau levelling osteotomy. A foam block ensured that the frontal plane of the tibia was horizontal. The toothed reduction handle was secured in place using a table-bound friction arm (Manfrotto, Videndum Media Distribution, Woodcliff Lake, NJ). The pes was in contact with the flat panel detector. The radiographic beam was centred on and parallel to the mechanical axis of the tibia. One PDHB view had no rotational stress. One PDHB view was acquired with internal rotational stress, and one with external rotational stress. Rotational stress was applied using a nylon rope that connected a 4.5-kg weight to the distal aspect of the metatarsal bones using a pulley.

### Assessment of Implant Orientation and Joint Stability

Radiographs were imported into radiographic viewing software (OrthoView, version 6.6.8, Materialise, Plymouth, MI) and were read by a single observer (M.M.Z.). Radiographs were calibrated for size using a 25-mm spherical marker. To confirm inclusion, a circle was fitted to the tibial cochlear groove on the ML view. The circle diameter was measured. The width of the trochlea of the talus was measured on the CC view. Limbs were included if the cochlear circle diameter was ≥13.0 mm and ≤15.0 mm, and the talar trochlear width was ≥16.0 mm, based on manufacturer recommendations for 14-mm cTAR implants.

**Fig. 1:**
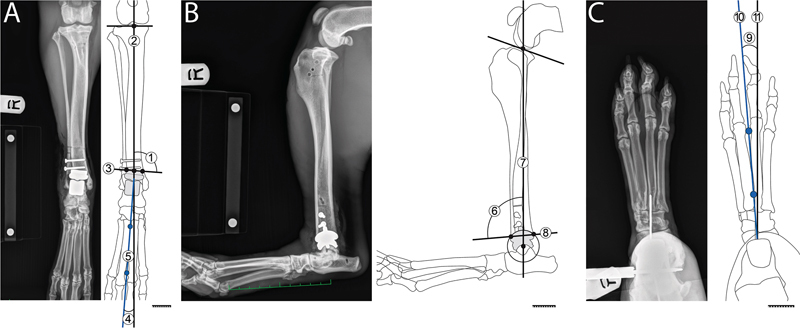
On the caudocranial radiographic view ( **A** ), the radiographic assessment of implant orientation after canine total ankle replacement included the mechanical medial distal tibial angle (1) between the mechanical axis of the tibia (2) and a line parallel to the proximal aspect of the tibial canine total ankle replacement component (3). Assessment of limb orientation consisted of measurement of the mechanical cranial distal tibial angle (4) between the mechanical axis of the metatarsal bones (5) and tibia (2). On the mediolateral view ( **B** ), implant orientation was the mechanical cranial distal tibial angle (6) between the mechanical axis of the tibia (7) and the tibial canine total ankle replacement component (8). On the proximodistal horizontal beam (skyline) view ( **C** ), the limb orientation angle (9) was the angle between a line fitted to the space between the third and fourth metatarsal bones (10) and a line parallel to a craniocaudal Kirschner wire inserted in the tibial crest (11). Caudocranial and proximodistal stress views were acquired with varus/valgus stress and internal/external rotational stress, respectively. For the dog shown, the mechanical medial distal tibial angle was 98.0 degrees, the mechanical cranial distal tibial angle was 92.1 degrees and the angle between the tibia and pes was 4.1 degrees of valgus.

**Fig. 2:**
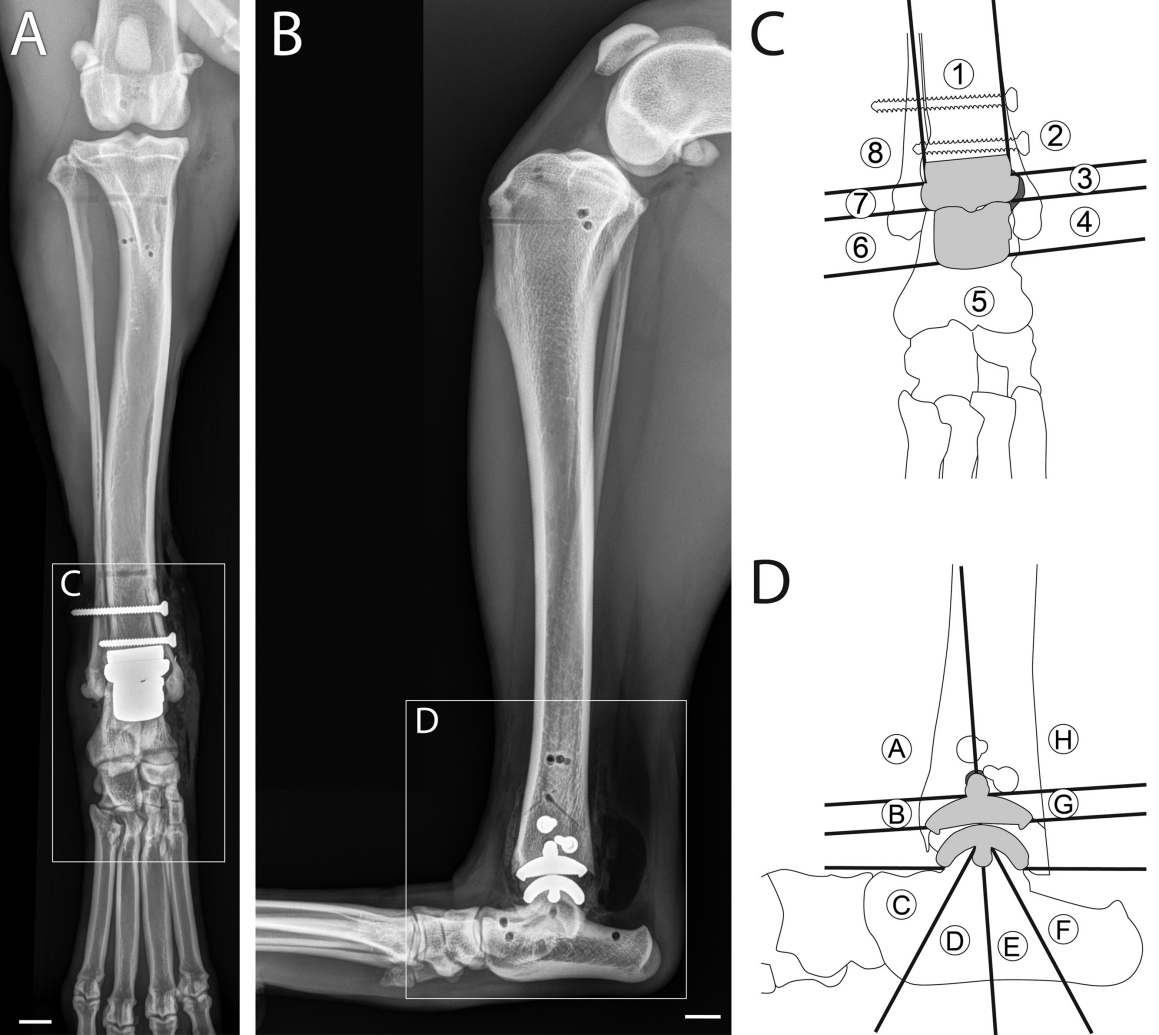
The width of bone–implant gaps was measured on caudocranial views ( **A** ) in five zones around the tibial component and three zones around the talar component ( **C** ) numbered clockwise from the top and on mediolateral views ( **B** ) in four zones around the tibial component and four zones around the talar component labeled counterclockwise (A–H) from top ( **D** ). Bone–implants gap width ranged from 0.32 to 1.66 mm on the caudocranial view and from 0.27 to 0.74 mm on the mediolateral view. Scale bar = 1 cm.

For radiographs acquired before cTAR, the mechanical medial proximal tibial angle and mechanical medial distal tibial angle (mMDTA) were measured on the CC view using a previously reported method. 11 The mechanical cranial distal tibial angle (mCrDTA) was measured on the ML view using a previously reported method. 13 For radiographs acquired after cTAR, the proximomedial and proximolateral edges of the cTAR tibial component were used to calculate the mMDTA on the CC view and the proximocranial and proximocaudal edges of the cTAR tibial component were used to calculate the mCrDTA on the ML view ( Fig. 1 ). Bone–implant interfaces were evaluated using zonal analysis using a previously reported method described for canine total hip replacement and used in canine total elbow replacement. 10 14 Bone–implant gaps were measured at 3 locations in 5 tibial and 3 talar zones on the CC view and 4 tibial and 4 talar zones on the ML view, for a total of 48 gap measurements in each operated limb. On the CC view, clockwise from dorsal, zones included the proximal (zone 1), proximomedial (2) and medial aspects (3) of the tibial component, the medial (4), distal (5) and lateral aspects (6) of the talar component, and the lateral (7) and proximolateral aspects (8) of the tibial component ( Fig. 2 ). On the ML view, counterclockwise from dorsal, zones included the cranial half of the tibial expansion post (zone A), the cranial aspects of the tibial (zone B) and talar components (zone C), the cranial (zone D) and caudal halves (zone E) of the talar expansion post, the caudal aspect of the talar (zone F) and tibial components (zone G) and the caudal half of the tibial expansion post (zone H).

Tarsal flexion was measured on the flexed ML view as the acute angle between the mechanical axis of the tibia and a line parallel to the dorsal surface of the third and fourth metatarsal bones. 15 To evaluate angular laxity, pes orientation was calculated from the CC view as the angle formed by the mechanical axis of the tibia and pes ( Fig. 1 ). 11 The mechanical axis of the pes was a line bisecting the space separating the third and fourth metatarsal bones. Varus laxity was calculated by subtracting the varus angle from the neutral CC view angle. Valgus laxity was calculated by subtracting the neutral CC angle from the valgus angle. Angular laxity was calculated by subtracting varus laxity from valgus laxity.

To evaluate rotational laxity, pes rotational orientation was calculated on the PDHB view as the angle formed by the craniocaudal axis of the tibia, marked by the Kirschner wire, and the mechanical axis of the pes, calculated as described above. Internal rotational laxity was calculated by subtracting the pes orientation from the internal rotational stress PDHB view from the pes orientation of the neutral PDHB view. External rotational laxity was calculated by subtracting the rotational pes orientation of the neutral PDHB view from the external rotational stress PDHB view. Rotational laxity was calculated by subtracting the pes rotational orientation of the internal rotational stress PDHB view from the external rotational stress PDHB view. Joints were considered stable if postimplantation angular or rotational laxity was increased by 9.4 degrees or less relative to preimplantation angular or rotational laxity. 12

### Approach

**Fig. 3:**
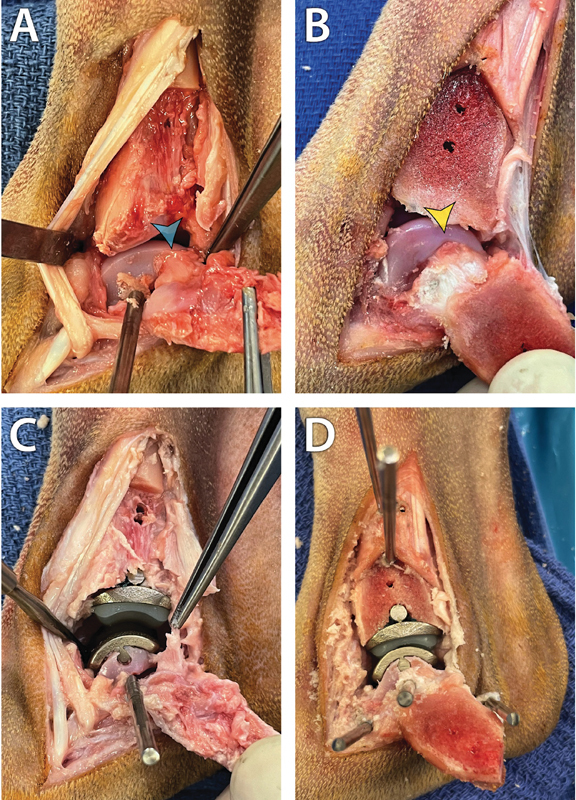
Implantation of the canine total ankle replacement prosthesis can be done using a lateral ( **A, C** ) or medial ( **B, D** ) approach. In this 40-kg dog, the insertion footprint of the short collateral ligament appeared closer to the caudoproximal edge of the trochlea of the talus on the lateral side of the joint (blue arrowhead in **A** ) than on its medial side (yellow arrowhead in **B** ). Damage to the insertion of the short collateral was common after a lateral approach (tissue grasped by the forceps in **C** ) but was not observed after a medial approach ( **D** ).

For the medial approach to the talocrural joint, the dog was positioned in lateral recumbency with the operated limb lowest. 6 Skin and subcutaneous tissues were incised on the medial aspect of the distal aspect of crus and tarsus. A parasagittal malleolar osteotomy was made using a 0.44-mm-thick blade on a sagittal saw ( Fig. 3 ). The proximodistal osteotomy length (distance from the apex of the chevron to the distal aspect of the medial malleolus) was equal to the ML width of the distal aspect of the tibia and fibula. The osteotomy depth reached the medial aspect of the talus. Depth was confirmed using fluoroscopy (TAU 2020, Orthoscan, Scottsdale, AZ). Care was taken to avoid damage to the medial collateral ligaments. Two 2.0-mm diameter holes were drilled in the osteotomized fragment before completion of the osteotomy. The osteotomized fragment was retracted distally and caudally.

The centre of rotation (COR) of the tarsocrural joint, located at the centre of the body of the talus, was identified and drilled with a 2.0-mm drill bit from medial to lateral. The talocrural joint was flexed and extended to confirm that the transtalar drill bit was coaxial with the ankle COR. The COR post was inserted in the talar hole. The alignment plate was placed over the COR post. The tibial component of the plate was aligned with the long axis of the tibial diaphysis, without placing torque on tissues or subluxating the ankle joint. The alignment plate was secured to the tibia using 2.8-mm pins inserted into metal sleeves. The pins were secured to the metal sleeves with set screws, and the metal sleeves were secured to the alignment plate with set screws. The articular surface of the talus was visualized in the window of the alignment plate. The tarsocrural joint was flexed to align the talar neck with a mark on the alignment plate. After that alignment, the tarsal angle was 95 to 100 degrees. Two 2.8-mm threaded pins were placed into the calcaneus and talus and secured to the alignment plate using metal sleeves and set screws. The drilling plate was placed on the alignment plate. A 4.5-mm tibial hole and a 3.5-mm talar hole were drilled from medial to lateral. Articular surfaces were removed using a mill through the alignment plate window. The milling arm was placed on the COR post with a 7.0-mm milling bit. Initial milling depth, controlled using a drill collar, was 2 mm shallower than a trial cTAR implant. Milling was started on the cranial aspect of the joint by plunging the mill to a depth of 2 to 3 mm, bringing the mill up, rotating the milling arm by approximately half of the mill diameter, and repeating the operation along the curvature of the milled area. The process was repeated four or five times, at increasing depths. The bone bed was lavaged, and the milled bone was removed. Milling was repeated with an 8.0-mm milling bit using the technique described above. The trial cTAR implant was inserted. Its depth relative to the medial aspect of the talus and medial tibial surface was evaluated. The milling process was repeated when increased depth was needed. The small bridges of intact bone remaining between milled surfaces and expansion post holes were cut using the rib breaker. A cTAR training prosthesis was secured to the inserter, aligned with the tibial and talar fixation posts and inserted into the prepared joint space using a mallet. The inserter was removed from the prosthesis. The pins, pin sleeves, alignment plate and COR post were removed. The medial malleolus was reattached using two 2.7-mm cortical screws placed in lag fashion through the previously drilled holes. Subcutaneous tissues were closed with 3–0 monofilament absorbable suture. Skin was closed with 3–0 nylon.

**Fig. 4:**
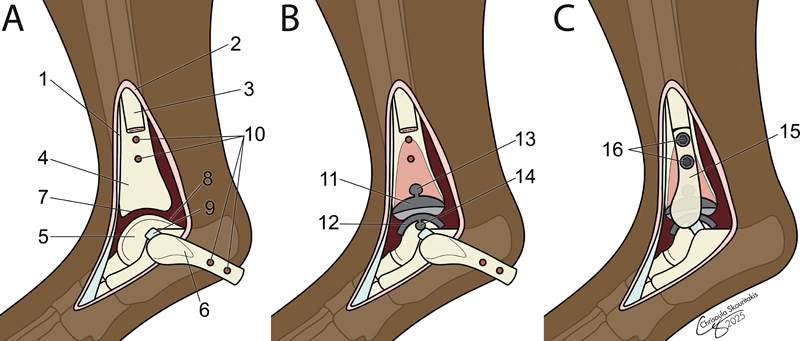
The lateral surgical approach to the tarsocrural joint ( **A** ) for implantation of the canine total ankle replacement prosthesis included cranial retraction of the tendons of the fibularis brevis and longus (1) and caudal retraction of the crural fascia (2), exposing the fibula (3), tibia (4) and talus (5). Once the osteotomized fibular fragment (6) was retracted, the lateral edge of the cochlea of the tibia (7) and lateral ridge of the trochlea of the talus (8) were visible. The insertion site of the short lateral collateral (9) was adjacent to 8. Two holes were predrilled (10) for stabilization of 6. After canine total ankle replacement implantation ( **B** ), the tibial (11) and talar components (12) were visible. Each component had an expansion post (13 and 14). Closure ( **C** ) included reduction of the fibular fragment (15) and stabilization using two 2.7-mm bone screws (16). Original illustration by: © Chrisoula Toupadakis Skouritakis, PhD.

For the lateral approach, the dog was positioned in lateral recumbency with the limb being implanted with a cTAR prosthesis uppermost. Skin and subcutaneous tissues were incised on the lateral aspect of the distal portion of the crus and tarsus, avoiding tendons and ligaments ( Fig. 4 ). A fibular osteotomy was made using a 0.44-mm-thick blade on a sagittal saw. The proximodistal length of the fibular segment was equal to the ML width of the malleoli. Two 2.0-mm holes were made in the osteotomized fragment before completion of the osteotomy. The proximal aspect of the fibular segment was elevated from the lateral aspect of the tibia to allow the fibular segment to be reflected distally and caudally, preserving the lateral collateral ligaments. Placement of the COR post, drilling, milling, assessment milling depth and implant insertion were performed using a method identical to the medial approach but working from lateral to medial. The lateral malleolus was reattached using two 2.7-mm screws placed in lag fashion. Subcutaneous tissues and skin were closed in a manner similar to that described for the medial approach. The joints were inspected for the presence of iatrogenic damage. Surgical obstacles were recorded.

### Statistical Analysis

Analyses were done using statistical software (SAS version 9.4; SAS Institute, Cary, NC). The approach (medial/lateral) and measurements (mMDTA, mCrDTA, flexion, varus angle, varus laxity, valgus laxity, frontal plane laxity, internal rotation angle, internal rotation laxity, external rotation laxity, rotational laxity before and after cTAR) were compared using an analysis of variance with repeated measures. Normality was evaluated using the Shapiro–Wilk test. Data were considered normally distributed when *W* > 0.90 and *p* > 0.05. Data that were not normally distributed were rank transformed before the analysis of variance. Pairwise comparisons were made using Tukey's honest significant difference *post hoc* tests. Significance was set at *p* < 0.05.

## Results

One dog was excluded because of the presence of a 31-degree valgus deformity of the distal aspect of the right tibia. Five dogs (10 limbs) were included in the study. The median dog weight was 29.7 kg (range, 26.0–45.0 kg). Osteoarthritis was not observed in any tarsal joint.

Prosthetic component orientation and tarsocrural laxity before and after cTAR are reported in Table 1 . Before implantation, valgus laxity was 1.8 degrees smaller in limbs operated using a lateral approach (2.8 degrees) than a medial approach (4.6 degrees, *p* = 0.026). Significant differences among limbs that underwent cTAR using a lateral and medial approach were not identified for other parameters of tibial geometry, tarsal motion, angular laxity or rotational laxity ( *p* ranging from 0.449 to 1.000).

**Table 1:** Mean ± standard deviation (median, range) prosthetic component orientation and tarsocrural laxity before and after total ankle replacement performed using a lateral or medial approach in five dogs weighing 26.0 to 45.0 kg

Parameter	Lateral approach	Medial approach
Preimplantation measurements		
Flexion (degrees)	39.0 ± 10.6 (38.4, 24.6–54.1)	38.0 ± 13.9 (30.7, 26.2–58.6)
Mechanical medial proximal tibial angle (degrees)	89.9 ± 1.3 (90.4, 88.0–91.2)	90.8 ± 2.4 (90.1, 88.1–93.4)
Mechanical medial distal tibial angle (degrees)	95.9 ± 3.0 (96.7, 90.7–98.1)	96.6 ± 3.2 (96.6, 91.4–99.7)
Mechanical cranial distal tibial angle (degrees)	87.2 ± 3.1 (87.2, 83.9–92.1)	86.6 ± 4.5 (84.6, 82.8–93.8)
Pes valgus a (degrees)	3.5 ± 0.7 (3.7, 2.3–4.1)	3.2 ± 3.1 (4.8, −1.0–6.1)
Valgus laxity a (degrees)	2.8 ± 0.9 d (2.7, 1.9–3.8)	4.6 ± 1.2 e (5.3, 2.8–5.5)
Varus laxity a (degrees)	−7.1 ± 1.8 (−7.2, −8.8 to −4.4)	−7.0 ± 3.1 (−6.9, −10.9 to −2.2)
Angular laxity b (degrees)	9.8 ± 2.0 (9.1, 8.1–12.6)	11.6 ± 3.2 (10.8, 7.7–16.4)
Pes rotation a (degrees)	3.7 ± 4.4 (0.5, 0.3–8.9)	5.0 ± 2.5 (6.3, 2.1–7.9)
Internal rotational laxity a (degrees)	−17.2 ± 2.9 (−16.7, −20.7 to −13.3)	−19.2 ± 5.4 (−16.4, −25.9 to −15.3)
External rotational laxity a (degrees)	5.3 ± 3.9 (4.0, 3.1–12.2)	4.9 ± 3.0 (5.4, 0.4–7.7)
Rotational laxity b (degrees)	22.5 ± 6.1 (20.9, 17.3–32.9)	24.0 ± 4.8 (22.7, 18.1–29.8)
Postimplantation measurements		
Flexion (degrees)	39.3 ± 11.7 (35.3, 28.0–55.8)	45.6 ± 11.4 (43.1, 30.3–61.7)
Mechanical medial distal tibial angle c (degrees)	91.7 ± 2.8 (92.8, 87.1–94.1)	87.8 ± 3.9 (86.8, 82.7–93.1)
Mechanical cranial distal tibial angle c (degrees)	95.7 ± 7.3 (96.2, 86.8–106.1)	97.5 ± 5.6 (99.1, 88.7–102.7)
Pes valgus a (degrees)	4.4 ± 2.0 (5.1, 2.1–6.5)	1.5 ± 5.5 (1.0, −6.1–9.2)
Valgus laxity a (degrees)	7.2 ± 5.0 (6.5, 2.7–15.2)	5.0 ± 3.4 (4.4, 0.9–9.5)
Varus laxity a (degrees)	−8.3 ± 3.7 (−8.0, −13.3 to −3.3)	−4.5 ± 2.7 (−5.8, −7.6 to −1.4)
Angular laxity b (degrees)	15.5 ± 6.7 d (18.2, 6.0–22.3)	9.5 ± 4.0 e (9.2, 4.2–15.4)
Pes rotation a (degrees)	8.2 ± 6.3 (7.6, 0.5–17.3)	2.8 ± 1.7 (2.2, 1.1–5.4)
Internal rotational laxity a (degrees)	−14.9 ± 9.7 (−11.7, −26.5 to −2.7)	−13.7 ± 7.0 (−12.7, −21.9 to −4.7)
External rotational laxity a (degrees)	10.7 ± 8.8 (10.8, −0.9–22.7)	11.6 ± 5.9 (11.8, 5.4–20.6)
Rotational laxity b (degrees)	25.6 ±12.9 (25.1, 9.5–45.6)	25.4 ± 9.2 (24.5, 17.3–39.9)

a Positive numbers represent valgus and external rotation.

b Angular (frontal plane) laxity was measured in tarsal extension and rotational (transverse plane) laxity was measured with the tarsus at approximately 90 degrees.

c Alignment of the tibial component of the canine total ankle replacement prosthesis.

d,e Within a row, mean values with different superscript letters differ statistically among approaches ( *p* < 0.05).

Several intraoperative obstacles were recorded. During the lateral approach, retraction of the osteotomized fibular fragment was challenging in three dogs. In two of these dogs, the COR placement was slightly proximal because of incomplete reflection of the osteotomized fragment. This led to slightly proximal milling in one dog: A narrow band of cartilage remained in the centre of the talar trochlear groove after milling. Transection of the cranial and caudal tibiofibular ligaments was deemed required to fully reflect the osteotomized fragment in one of these three dogs. Insertion of the calcaneal post was considered challenging in one dog. The mill interfered with the insertion site of the short lateral collateral ligament in four dogs, leading to damage to the short lateral collateral ligament ( Fig. 3 ). Damage was mild in three dogs, and moderate in one dog. The dogs weighed 26.0, 29.3, 40.0 and 45.0 kg. The cTAR prosthesis protruded slightly in three dogs. Minor reaming of the osteotomized fibular fragment was required before reduction in these three dogs.

During the medial approach, the caudal cut of the medial malleolar osteotomy was deeper than anticipated in one dog, despite the use of fluoroscopy. Partial milling of the fibula was required to mill to depth for full cTAR implantation in one dog. The placement of the malleolar reduction screws was challenging in three dogs. In one of these dogs, the two malleolar screws did not achieve stable reduction. One screw was redrilled, and the other was replaced with a 1.40-mm Kirschner wire. The cTAR prosthesis interfered with fragment reduction in two of these dogs. The two screws were redrilled in one dog, and the distal screw was redrilled in the other dog. Additional iatrogenic damage was not observed.

When comparing postimplantation to preimplantation measurements after a lateral approach, mean mCrDTA was 8.5 degrees larger after implantation (95.7 degrees) than before (87.2 degrees, *p* = 0.023), mean valgus laxity was 4.4 degrees larger after implantation (7.2 degrees) than before (2.8 degrees, *p* = 0.035), and mean external rotational laxity was 5.4 degrees larger after implantation (10.7 degrees) than before (5.3 degrees, *p* = 0.014, Table 1 ). Significant differences were not identified for other measurements collected before and after a lateral approach ( *p* ranging from 0.058 to 0.975). When comparing postimplantation to preimplantation measurements after a medial approach, the mean mMDTA was 8.8 degrees smaller after implantation (87.8 degrees) than before (96.6 degrees, *p* = 0.001), the mean mCrDTA was 10.9 degrees larger after implantation (97.5 degrees) than before (86.6 degrees, *p* = 0.005), and mean external rotational laxity was 6.7 degrees larger after implantation (11.6 degrees) than before (4.9 degrees, *p* = 0.014). Significant differences were not identified for other measurements collected before and after a medial approach ( *p* ranging from 0.155 to 0.833). When comparing measurements after a lateral or a medial approach, mean angular laxity was 6.0 degrees larger after a lateral approach (15.5 degrees) than a medial approach (9.5 degrees, *p* = 0.044). Significant differences were not identified for other measurements collected after a lateral or a medial approach ( *p* ranging from 0.059 to 0.962). Median bone–implant gaps (range) after a lateral approach (0.46 mm, 0.20 to 1.32 mm) were similar to gaps after a medial approach (0.44 mm, 0.18 to 2.39 mm). After a lateral approach, 40% of gaps measured <0.5 mm and 96% measured <1.0 mm and after a medial approach, 35% of gaps measured <0.5 mm and 96% measured <1.0 mm. Gaps did not differ statistically for 43 of 48 paired zone measurements ( *p* ranging from 0.100 to 0.987), were smaller after a medial approach for four paired zone measurements ( *p* ranging from 0.016 to 0.037) and were larger after a medial approach for one paired zone measurement ( *p* = 0.046).

## Discussion

In the current study, cTAR was successfully implanted using a lateral approach. Implant orientation, limb orientation and joint stability were similar before and after implantation and were similar when a lateral or medial approach was used. The orientation of the cTAR prosthesis differed slightly from the orientation of the native joint in the frontal and sagittal planes. A slight increase in angular laxity was observed after cTAR implantation using a lateral approach.

Prosthetic alignment and limb alignment differed minimally after lateral or medial cTAR implantation. Differences among all means were <4 degrees. We accepted the hypothesis that cTAR implantation using a lateral approach was accurate. Prosthetic and limb alignment after lateral cTAR implantation differed minimally compared with preimplantation alignment: Differences among all means were <2 degrees, except for the mCrDTA that increased by 8.5 degrees. The postimplantation increase in mCrDTA indicated that the cTAR was placed in a slightly more declined position compared with the alignment of the distal aspect of the tibia before implantation. Since the cTAR prosthesis is a cartridge implant where both components are aligned and implanted simultaneously, the mCrDTA of the tibial and talar components matched. Prosthetic or anatomical impingement was not observed during implantation. The mMDTA decreased after implantation using a medial and lateral approach. The approximately 9-degree decrease after a medial approach was significant and the approximately 4-degree decrease after a lateral approach was not significant. For cTAR prostheses implanted using a medial and a lateral approach, the decrease in mMDTA resulted from the fact that the mean mMDTA before implantation was approximately 96 degrees (6 degrees of valgus), while the target mMDTA after implantation was 90 degrees, since implant alignment was perpendicular to the mechanical axis of the tibia. Placement of the cTAR prosthesis with an mMDTA of 90 degrees required the distal and caudal retraction of the malleolar segment. Incomplete retraction of the malleolar segment would lead to a tilted alignment plate with varus cTAR orientation after a medial approach and valgus cTAR prosthesis orientation after a lateral approach. Accordingly, cTAR prostheses implanted using a medial approach had a slight varus (mean, 2.2 degrees) and cTAR prostheses implanted using a lateral approach had a slight valgus (mean, 1.7 degrees), each most likely resulting from slight interference of the malleolar segment with the alignment plate. Subjectively, the bulk of the lateral malleolar fragment was less than the bulk of the medial malleolar fragment.

We accepted the hypothesis that lateral cTAR implantation leads to a stable tarsocrural joint.

To test the stability of the short medial and lateral collateral ligaments and long medial and lateral collateral ligaments, tarsocrural motion in the transverse plane was measured with the joint held at 90 degrees and tarsocrural motion in the frontal plane was measured with the joint in extension. After a lateral approach, lateral collateral laxity was not detected in tarsocrural flexion or extension, but mild laxity was detected in flexion (+5.4 degrees of external rotational laxity relative to preimplantation motion) and extension (+4.4 degrees of valgus laxity). After a medial approach, medial collateral ligament laxity was also detected in flexion (+6.7 degrees of external rotational laxity) but not in extension (+0.4 degrees of valgus laxity). The cause of that mild postimplantation laxity is not known. It could result from the soft tissue dissection or a mild loss of tissue tension after cTAR implantation.

Notably, damage to the short lateral collateral ligament was observed after milling in four of five lateral approach surgeries. Damage did not appear to result from an excessively small talus size, since it was observed in two dogs weighing 40.0 and 45.0 kg. Damage was possibly due to the location of the short collateral ligament insertion footprint. That footprint appeared closer to the talar articular surface on the lateral side than the medial side. The function of the medial and lateral short collateral ligaments is asymmetric. In dog cadavers, isolated short lateral collateral ligament transection causes varus, but isolated short medial collateral ligament does not cause valgus. 16 Collateral ligaments of the ankle are also asymmetric in the human ankle; five ligaments are described on the lateral side and six on the medial side, with individual variation. 17 18 Because of its insertion close to the caudodorsal edge of the talar trochlea, the short lateral collateral ligament could also be at risk of damage during cTAR implantation using a medial approach. That potential damage was not evaluated in the current study. The absence of internal rotational laxity after lateral and medial cTAR implantation suggests that no loss of function of the short lateral collateral ligaments occurred. Damage to the insertion of the short lateral collateral ligament was possibly associated with an excessively caudal or proximal position COR post placement. Subjectively, drilling for COR post placement was more challenging on the lateral aspect of the talus than on its medial aspect. This is because the COR of the tarsus appeared to be immediately dorsal to the space separating the talus and the sustentaculum tali of the calcaneus. Slightly ventral placement of the drill bit led to slippage between the talus and calcaneus. The position of the anatomical COR of the talocrural joint has been reported to vary among dogs within a breed (Greyhound) and most likely varies among dog breeds. 19 Future research is needed to describe the insertion footprint of the lateral and medial collateral ligaments and the location of the COR of the talus on its lateral and medial aspects. Also, strategies to ensure accurate COR post placement and minimize damage to the short collateral ligaments should be explored.

Difficulties were encountered during stabilization of malleolar osteotomies after both lateral and medial approaches. In clinical patients, the malleolar osteotomies created for total ankle replacement are generally stabilized using bone plates or can be stabilized with bone screws with an outer diameter measuring 30% of the width of the osteotomized malleoli. 6 In the current study, relatively small screws were used for malleolar fragment stabilization to maximize the visibility of the prosthetic components and bone–implant interfaces. These screws provided sufficient stability to perform the non-destructive tests. Future research is needed to evaluate the convenience, stability and healing of various malleolar stabilization methods for medial and lateral cTAR approaches.

Gaps at bone–implant interfaces after lateral or medial cTAR implantation were small. Most gaps were <1.0 mm. Bone–implant interfaces were excellent, considering that surgeries were performed with training implants rather than clinical implants. Training implants have a looser tolerance than clinical implants. They also lack expansion posts. Those posts enhance immediate implant fixation and likely decrease bone–implant gaps. The accuracy of implantation of cartridge total joint prostheses has been evaluated in the dog elbow. 10 In that study, implanting the total elbow components using a lateral approach was also accurate (mean, 2.7 degrees of varus) and differed minimally from components implanted using a medial approach (mean, 1.7 degrees of varus). While the geometry and motion of the elbow and tarsus have clear similarities, key differences exist. While the COR of the elbow is in the centre of the humeral condyle, the COR of the tarsus is located relatively close to the plantar edge of the talus. Therefore, more caution is required when drilling the COR post pilot hole in the tarsus compared with the humerus to avoid slipping off the bone, particularly during a lateral approach, because the talus is proximodistally shorter on its lateral aspect than on its medial aspect. Also, more muscles cover the elbow than the tarsus, particularly on the medial aspect of the joint. While the lateral approach to the elbow offers the key advantage of minimizing the need to retract larger muscles over the medial approach to the elbow, the lateral approach to the tarsocrural joint does not appear to offer such advantages. 10 The accuracy of cTAR implantation in the current study is similar to the accuracy of placement of total ankle replacement prostheses in humans, where malalignment in the coronal (frontal) and sagittal planes is usually <2 degrees. 20 Patient-specific guides have been used in an attempt to increase the accuracy of total ankle replacement in humans, but it is unclear whether accuracy increases when patient-specific guides replace conventional instrumentation. 20 Patient-specific guides for epicondylar osteotomy and COR post placement have been described for total elbow replacement in dogs. 10 Considering that the precision of the osteotomy or COR post placement was suboptimal in 3 of 10 procedures in the current study, patient-specific guides may be beneficial to the precision of cTAR implantation.

Based on the findings of the current study, a lateral approach for insertion of the cTAR prosthesis appears to represent an acceptable option with, subjectively, additional challenges relative to a medial approach that include the COR post placement and potential damage to the insertion site of the short lateral collateral ligament. A lateral approach is likely a valid option when a medial approach is particularly challenging or carries risks, including abnormal soft tissue coverage, a flared or deformed medial malleolus that would complicate a medial malleolar osteotomy, or the presence of metal implants. For limbs in the current study, tibiofibular synostoses were not observed. To our knowledge, tibiofibular synostosis has not been reported in the dog, but it has been described in the human crus as a developmental problem or as the result of tibiofibular syndesmosis using a non-absorbable braided suture. 21 22 If tibiofibular synostosis was present, a tibiofibular osteotomy would be required to elevate the fibular fragment.

The current study had limitations. The sample size was small due to logistical limitations. The small sample size increased the likelihood of type II statistical error. Subjectively, near-identical findings after lateral and medial approaches for most parameters suggest that small sample size did not prevent the assessment of accuracy and joint stability after cTAR. Notably, however, several standard deviations were large, indicating large differences among dogs. Standard deviations were largest for rotational tarsocrural laxity after a lateral (13 degrees) and medial approach (9 degrees). The tarsal joints in the current study did not have osteoarthritis. The influence of osteophytes and articular fibrosis on the accuracy and stability of the tarsus after cTAR prostheses implanted using a medial or lateral approach is not known. Postimplantation tarsocrural extension was not measured. However, the placement accuracy of the tibial component in the sagittal plane was high and did not differ among approaches.

We concluded from the current study that cTAR can be performed using a lateral approach, albeit with challenges due to COR post placement and potential damage to the short lateral collateral ligament, resulting in a properly oriented tarsocrural joint that is rotationally stable and has slight angular laxity. A lateral approach may be considered when a medial approach is deemed to carry increased risks.
